# Oesophageal atresia

**DOI:** 10.1186/1750-1172-2-24

**Published:** 2007-05-11

**Authors:** Lewis Spitz

**Affiliations:** 1 Department of Paediatric Surgery, Institute of Child Health, University College, London, UK; 2 Department of Paediatric Surgery, Great Ormond Street Hospital for Children, London, UK

## Abstract

Oesophageal atresia (OA) encompasses a group of congenital anomalies comprising of an interruption of the continuity of the oesophagus with or without a persistent communication with the trachea. In 86% of cases there is a distal tracheooesophageal fistula, in 7% there is no fistulous connection, while in 4% there is a tracheooesophageal fistula without atresia. OA occurs in 1 in 2500 live births. Infants with OA are unable to swallow saliva and are noted to have excessive salivation requiring repeated suctioning. Associated anomalies occur in 50% of cases, the majority involving one or more of the VACTERL association (vertebral, anorectal, cardiac, tracheooesophageal, renal and limb defects). The aetiology is largely unknown and is likely to be multifactorial, however, various clues have been uncovered in animal experiments particularly defects in the expression of the gene Sonic hedgehog (*Shh*). The vast majority of cases are sporadic and the recurrence risk for siblings is 1%. The diagnosis may be suspected prenatally by a small or absent stomach bubble on antenatal ultrasound scan at around 18 weeks gestation. The likelihood of an atresia is increased by the presence of polyhydramnios. A nasogastric tube should be passed at birth in all infants born to a mother with polyhydramnios as well as to infants who are excessively mucusy soon after delivery to establish or refute the diagnosis. In OA the tube will not progress beyond 10 cm from the mouth (confirmation is by plain X-ray of the chest and abdomen). Definitive management comprises disconnection of the tracheooesophageal fistula, closure of the tracheal defect and primary anastomosis of the oesophagus. Where there is a "long gap" between the ends of the oesophagus, delayed primary repair should be attempted. Only very rarely will an oesophageal replacement be required. Survival is directly related to birth weight and to the presence of a major cardiac defect. Infants weighing over 1500 g and having no major cardiac problem should have a near 100% survival, while the presence of one of the risk factors reduces survival to 80% and further to 30–50% in the presence of both risk factors.

## Disease name and synonyms

Oesophageal atresia with or without tracheooesophageal fistula, OA, TOF, TEF (American)

## Definition

Oesophageal atresia encompasses a group of congenital anomalies comprising an interruption of the continuity of the oesophagus combined with or without a persistent communication with the trachea.

## Epidemiology

Oesophageal atresia is a relatively common congenital malformation occurring in one in 2500–3000 live births. The overwhelming majority of cases of oesophageal atresia are sporadic/non-syndromic, although a small number within this non-familial group are associated with chromosomal abnormalities. Familial/syndromic cases of oesophageal atresia are extremely rare, representing less than 1% of the total. Oesophageal atresia is 2 to 3 times more common in twins [1].

## History

The first clearly documented case of oesophageal atresia, confirmed at post-mortem examination, was recorded by Thomas Gibson [2] in 1697, as follows: "About November 1696, I was sent for to an infant that would not swallow. The child seemed very desirous of food, and took what was offered it in a spoon with greediness; but when it went to swallow it, it was liked to be choked, and what should have gone down returned by the mouth and nose, and it fell into a struggling convulsive sort of fit upon it."

The next recorded case was almost 150 years later by Thomas Hill [3] in 1840 who "was called, in the night, to visit Dr Webster's family". The newborn infant "made no effort to swallow but immediately convulsed and the drink which had been given returned by mouth and nose, mixed with bloody mucus". He recommended that "gently stimulating the rectum would remove the difficulty", however, when an attempt was made to do so, there was "no vestige of an anus". Hill was the first to document an associated anomaly with oesophageal atresia.

Thomas Holmes [4] in 1869 was the first to suggest the possibility of operative treatment but he added "the attempt ought not, I think, be made".

In 1913, Richter [5] proposed an operative plan consisting of ligation of the tracheooesophageal fistula and anastomosis of the two ends of the oesophagus.

Lanman [6] was the first to perform an extrapleural repair in 1936. His patient lived for only three hours and in 1940 he reported his experience with 30 operative cases, all of whom died. He stated that "with greater experience, improved technique and good luck" success would soon be reported.

The early successes with oesophageal atresia were documented separately by Leven [7] and Ladd [8] in 1939 reporting the first survivors following staged repair with later oesophageal replacement. Cameron Haight [9] performed the first successful primary repair in 1941. His patient was an infant girl, 12 days old on admission who had been transported over a distance of 500 miles to Ann Arbor, Michigan. The operation *via *an extrapleural approach comprised ligation of the tracheooesophageal fistula and an end-to-end anastomosis. The child survive despite a stormy postoperative course with anastomotic leakage and stricture formation.

Thereafter the improvement in survival which followed was spectacular. Waterston *et al*. [10] reported a survival rate of 57.6% in 113 infants treated in the early 1950's. Willis Potts [11] in 1950 wrote "To anastomose the ends of an infant's oesophagus, the surgeon must be as delicate and precise as a skilled watchmaker. No other operation offers a greater opportunity for pure technical artistry". By the mid-1980's mortality had fallen to below 15% with some centres reporting success rates of over 90%.

## Classification

**Figure 1:**
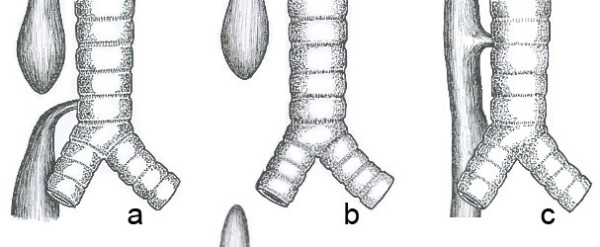
Common anatomical types of oesophageal atresia. a) Oesophageal atresia with distal tracheooesophageal fistula (86%). b) Isolated esophageal atresia without tracheooesophageal fistula (7%). c) H-type tracheooesophageal fistula (4%)

The original classification by Vogt [12] in 1929 is still used today. Ladd (1944) [8] and Gross (1953) [13] modified the classification, while Kluth [14] (1976) published an extensive "*Atlas of Esophageal Atresia" *which comprised 10 major types, each with numerous subtypes which is based on the original Vogt classification. It would appear to be more valuable to describe the anatomical anomaly rather than assign a label which may not be widely recognisable (Fig 1).

### 1. Oesophageal atresia with distal tracheooesophageal fistula (86% Vogt 111b. Gross C)

This is the most common variety in which the proximal oesophagus, which is dilated, and the muscular wall thickened ends blindly in the superior mediastinum at about the level of the third or fourth thoracic vertebra. The distal oesophagus, which is thinner and narrower, enters the posterior wall of the trachea at the carina or more commonly one to two centimetres more proximally in the trachea. The distance between the blind proximal oesophagus and the distal tracheooesophageal fistula varies from overlapping segments to a wide-gap. Very rarely the distal fistula may be occluded or obliterated leading to the misdiagnosis preoperatively of an isolated atresia.

### 2. Isolated oesophageal atresia without fistula (7%, Vogt 11, Gross A)

The proximal and distal oesophagus end blindly without any connection to the trachea. The proximal oesophageal segment is dilated and thick-walled and usually ends higher in the posterior mediastinum at around the second thoracic vertebra. The distal oesophagus is short and ends a variable distance above the diaphragm. The distance between the two ends will determine whether a primary repair is feasible (rarely) or a whether a delayed primary anastomosis or an oesophageal replacement should be performed. It is important to exclude a proximal tracheooesophageal fistula in these cases.

### 3. Tracheooesophageal fistula without atresia (4%, Gross E)

There is a fistulous connection between an anatomically intact oesophagus and trachea. The fistulous tract may be very narrow or 3–5 mm in diameter and is commonly located in the lower cervical region. They are usually single but two and even three fistulas have been described.

### 4. Oesophageal atresia with proximal tracheooesophageal fistula (2%, Vogt III and Gross B)

This rare anomaly needs to be distinguished from the isolated variety. The fistula is not at the distal end of the upper pouch but is sited 1–2 cm above the end on the anterior wall of the oesophagus.

### 5. Oesophageal atresia with proximal end distal tracheooesophageal fistula (<1%m Vogt IIIa, Gross D)

In many of these infants the anomaly was misdiagnosed and managed as proximal atresia and distal fistula. As a result of recurrent respiratory infections, investigations carried out revealed a tracheooesophageal fistula, previously mistaken for a recurrent fistula. With the increasing use of preoperative endoscopy (bronchoscopy and/or oesophagoscopy) early recognition of the "double" fistula is made and total repair performed at the initial procedure. If the proximal fistula is not identified preoperatively, the diagnosis should be suspected by a large gas leak emanating from the upper pouch during the fashioning of the anastomosis.

## Clinical description and diagnosis

Delaying the diagnosis until the infant presents with coughing and choking during the first feed is no longer acceptable in modern paediatric practice.

The diagnosis of oesophageal atresia may be suspected prenatally by the finding of a small or absent fetal stomach bubble on ultrasound scan performed after the 18^th ^week of gestation. Overall the sensitivity of ultrasonography is 42% but in combination with polyhydraminos the positive predictive value is 56% [15]. Polyhydraminos alone is a poor indication of oesophageal atresia (1% incidence). Available methods of improving the prenatal diagnostic rate include ultrasound examination of the fetal neck to view the blind-ending upper pouch [16] and to observe fetal swallowing and magnetic resonance imaging [17].

**Figure 2:**
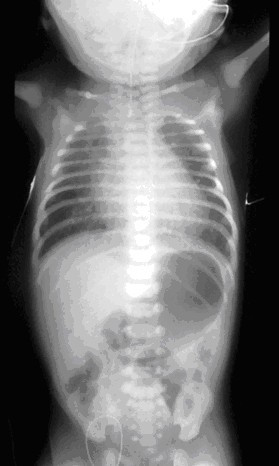
Plain X-ray of the chest and abdomen showing the radio-opaque tube in the blind upper oesophageal pouch. Air in the stomach indicates the presence of a distal tracheooesophageal fistula.

The newborn infant of a mother with polyhydramnios should always have a nasogastric tube passed soon after delivery to exclude oesophageal atresia. Infants with oesophageal atresia are unable to swallow saliva and are noted to have excessive salivation requiring repeated suctioning. At this stage, and certainly before the first feed, a stiff wide-bore (10–12 French gauge) catheter should be passed through the mouth into the oesophagus. In oesophageal atresia the catheter will not pass beyond 9–10 cm from the lower alveolar ridge. A plain X-ray of the chest and abdomen will show the tip of the catheter arrested in the superior mediastinum (T 2–4) while gas in the stomach and intestine signifies the presence of a distal tracheooesophageal fistula (Fig 2). The absence of gastrointestinal gas is indicative of an isolated atresia. A fine bore catheter may curl up in the upper pouch giving the false impression of an intact oesophagus or rarely it may pass through the trachea and proceed distally into the oesophagus through the fistula. The X-ray may reveal additional anomalies such as a "double bubble" appearance of duodenal atresia, vertebral or rib abnormalities.

### Associated anomalies

Over 50% of infants with oesophageal atresia have one or more additional anomalies [18]. The systems affected are as follows:

Cardiovascular 29%

Anorectal anomalies 14%

Genitourinary 14%

Gastrointestinal 13%

Vertebral/skeletal 10%

Respiratory 6%

Genetic 4%

Other 11%

There is an increased incidence of associated anomalies in pure atresia (65%) and a lower incidence in H-type fistula (10%).

The VATER association first described by Quan and Smith [19] in 1973 consists of a combination of anomalies including **v**ertebral, **a**norectal, **t**racheoo**e**sophageal and **r**enal or radial abnormalities. This association was later expanded as the VACTERL association to include **c**ardiac and **l**imb defects [18].

Other associations which may include oesophageal atresia are the CHARGE association (coloboma, heart defects, atresia choanal, retarded growth and development, genital hypoplasia and ear deformities), POTTER'S syndrome (renal agenesis, pulmonary hypoplasia, typical dysmorphic facies) and SCHISIS association (omphalocoele, cleft lip and/or palate, genital hypoplasia). Genetic defects associated with oesophageal atresia include Trisomy 21 and 18, and 13q deletion. Of the cardiac anomalies, the most common are ventricular septal defect and tetralogy of Fallot. Major cardiac malformations are one of the main causes of mortality in infants with oesophageal atresia[18,20].

The vertebralanomalies in oesophageal atresia are mainly confined to the thoracic region and are responsible for later development of scoliosis. The claim that the presence of 13 ribs is associated with long-gap atresia has not been substantiated. Of the gastrointestinal anomalies, the most frequently encountered are duodenal atresia and malrotation, while there is an increased incidence of pyloric stenosis. Miscellaneous anomalies include cleft lip and palate, omphalocoele, lung abnormalities, choanal atresia and hypospadias.

## Aetiology

The aetiology of oesophageal atresia is likely to be multifactorial and remains unknown.

### Embryology

The mechanism that underlie tracheooesophageal malformations are still unclear, however, the development of reproducible animal models of these anomalies has allowed detailed analysis of the various stages of faulty organogenesis. By contrasting these stages with normal development, it has been possible to identify key developmental processes that may be disturbed during embyogenesis.

It is generally accepted that the respiratory primordium appears as a ventral evagination on the floor of the post-pharyngeal foregut at the beginning of the fourth week of gestation and that the primitive lung buds are located at the caudal end of this evagination [21]. During a period of rapid growth, the ventrally placed trachea becomes separated from the dorsally placed oesophagus. One theory postulates that the trachea becomes a separate organ as a result of rapid longitudinal growth of the respiratory primordium away from the foregut [22]. An alternative theory is that the trachea initially grows as part of an undivided foregut and then becomes a separate structure as a result of a separation process that starts at the level of the lung buds and proceeds in a cranial direction [23]. This process is associated with a precise temporospatial pattern of expression of the key developmental gene *Sonic hedgehog *(*Shh*) and members of its signalling cascade. A precise ventral-to-dorsal switch in foregut *Shh *expression is itself propagated cranially, ahead of tracheooesophageal separation [24]. The separating foregut epithelium is marked by increased numbers of cells undergoing programmed cell death [25]. Theories of abnormal organogenesis reflect the theories of normal development and are largely based on evidence from the Adriamycin rat model of oesophageal atresia (OA) and tracheooesophageal fistula (TOF) and a more recently described mouse model [24,26,27]. In sporadic cases of oesophageal atresia, the likely cause is an insult that occurs during the narrow gestational window of tracheooesophageal organogenesis; this is the basis of the Adriamycin animal models. Most studies suggest that the primary defect is the persistence of an undivided foregut, either as a result of failure of tracheal growth [24] or failure of the already specified trachea to physically separate from the oesophagus [27]. An alternative theory suggests that atresia of the proximal oesophagus is the primary event and that the malformation results secondarily from the establishment of continuity between the trachea and the stomach/distal esophagus (tracheooesophageal fistula) [28].

The development of genetic models of tracheooesophageal malformations has provided an insight into the molecular mechanisms that underlie these defects. In the mouse, loss of function mutations of *Shh *and other members of its signalling pathway (*Gli2, Gli3 & Foxf1*) lead to tracheooesophageal malformations including OA/TOF [29]. In the *Shh *mutant, failure of tracheooesophageal separation is the underlying abnormality. In the Adriamycin model, failure of tracheooesophageal separation is associated with disturbance of the temporospatial pattern of *Shh *expression [24].

The presence of associated malformations could provide clues as to the possible aetiology of oesophageal atresia. Such malformations are present in 50% of cases and can occur in distinct patterns [18]. These are non-random associations rather than syndromes because the presence of anomalies in one system makes it more likely that defects exist in another. One of the best-described associations is the **VACTERL **association, which comprises **v**ertebral, **a**norectal**, c**ardiac, **t**racheo-o**e**sophageal, **r**enal and **l**imb abnormalities. The pattern of these associations is likely to be dictated by the timing of a possible insult that affects multiple morphogenetic events. In the rat model, Adriamycin exposure affects a number of systems broadly consistent with the VACTERL association [30]. The insult could act by transiently disturbing a specific developmental signalling pathway. In the mouse, loss of function mutations for genes of the *Shh *pathway lead to a spectrum of anomalies that is very similar to those of VACTERL, implicating the pathway in the embryogenesis of the malformations[31]. In the human, the evidence is less conclusive, with the *Shh *mutation leading to holoprosencephaly [32], a malformation not associated with oesophageal atresia. Tracheooesophageal fistula and other features of VACTERL have however been described in patients with a mutation for *GL13*[33].

Chromosomal abnormalities such as trisomies (18 and 21) [34] and deletions (22q11 and 17q22q23.3) [35,36] are known to be associated with oesophageal atresia and have been reported in up to 6% of patients who have associated malformations in other systems [37].

### Pathophysiology

The motility of the oesophagus is always affected in oesophageal atresia. The disordered peristalsis more commonly involves the distal oesophageal segment. Whether the motility disorder is primarily due to abnormal innervation as evidenced by an abnormality in neuropeptide distribution [38,39] or secondary to vagal nerve damage occurring during the surgical repair remains uncertain. The resting pressure in the whole oesophagus is significantly higher than in normal patients and the closing pressure of the lower oesophageal sphincter is reduced.

The trachea is also abnormal in oesophageal atresia. The abnormality consists of an absolute deficiency of tracheal cartilage and an increase in the length of the transverse muscle in the posterior tracheal wall [40]. When severe, these abnormalities result in tracheomalacia with collapse of the trachea over a 1–2 cm segment in the vicinity of the fistula.

## Genetic counselling

The overwhelming majority of cases of oesophageal atresia are sporadic/non-syndromic, although a small number within this non-familial group are associated with chromosomal abnormalities. Familial/syndromic cases of oesophageal atresia are extremely rare. Oesophageal atresia is 2 to 3 times more common in twins [1]. The overall risk of oesophageal atresia recurrence in a sibling of an affected child is about 1%.

## Management

### A. Preoperative

Once the diagnosis of oesophageal atresia has been established, the infant will need to be transferred from the place of birth to a regional paediatric surgical centre. A suction catheter, preferably of the double lumen type (Replogle catheter No.10 French gauge), is placed in the upper oesophageal pouch to suction secretions and prevent aspiration occurring during transfer. The infant is placed on its side in the portable incubator while monitoring the usual vital signs. Vascular access should be provided as a precautionary measure but intravenous fluid administration is not usually necessary if the condition has been diagnosed within a short period after birth and transfer is carried out within the first day of life.

The preterm infant with respiratory distress requires special attention. Clearly there is a need for endotracheal intubation and mechanical ventilation. In addition, there is the added risk of gastric over-distension and rupture of the stomach due to escape of respiratory gases down through the distal fistula into the stomach due to the increased pulmonary resistance. This sequence of events can be minimised by positioning the end of the endotracheal tube distal to entry of the tracheooesophageal fistula and by applying gentle low pressure ventilation.

Upon arrival at the Neonatal Surgical Centre, the diagnosis of oesophageal atresia must be confirmed.

All infants with oesophageal atresia should have an echocardiogram prior to surgery. The ECHO will define any structural anomaly of the heart or great blood vessels and occasionally may indicate a right-sided aortic arch which occurs in 2.5% of cases [41,42]. In these cases, a magnetic resonance imaging study is the method of choice for accurate confirmation of the diagnosis of the right aortic arch and will determine the side of approach for the operative repair. In around 25% of infants with tetralogy of Fallot, the aortic arch will be on the right side. The infant with a major cardiac anomaly resulting in severe cyanotic episodes will need to undergo a shunting procedure prior to correction of the oesophageal atresia. For example, in complicated tetralogy of Fallot, a Blalock-Taussig systemic-to-pulmonary artery shunt will alleviate the cyanotic attacks and permit repair of the oesophageal atresia to take place a day or two later [43]. Congestive cardiac failure will necessitate medical management in the first instance.

### B. Risk categorisation and prognosis (outcome)

In 1962, Waterston *et al*. [10] proposed a classification of infants born with oesophageal atresia into three groups "with different chances of survival". The classification based on birth weight, associated anomalies and pneumonia comprised:

**Group A **Over 5 1/2 lb (2500 g) birth weight and well

**Group B **1. Birth weight 4–5 1/2 lb (1800–2500 g) and well

2. Higher birthweight, moderate pneumonia and congenital anomaly.

**Group C **1. Birth weight under 4 lb (1800 g).

2. Higher birth weight and severe pneumonia and severe congenital anomaly.

This classification was applied to 113 cases treated at Great Ormond Street Hospital from 1951 to 1959. Of 38 infants in Group A, all but two survived (95%), of 43 in Group B 29 (68%) survived while only two (6%) of the 32 in Group C survived.

During the subsequent 40 years there has been a steady improvement in the overall survival rate due to early diagnosis and prompt referral, improvements in preoperative care and diagnosis and treatment of associated anomalies, advances in anaesthetic techniques and sophisticated neonatal intensive care. Applying the Waterston classification to a series of 357 infants with oesophageal atresia treated at Great Ormond Street from 1980 to 1992 [44], the results were Group A, 153 of 154 survived (99%), 72 of 76 in Group B survived (95%) and 101 of 142 in Group C survived (71%). It became obvious that a new risk classification system was needed which was more relevant to the modern era. The new risk classification concerned birth weight and associated cardiac malformations which were previously identified as being responsible for most of the mortality.

The Spitz classification [44] for survival in oesophageal atresia is:

**Group I **Birth weight over 1500 g **with no **major cardiac anomaly.

**Group II **Birth weight less than 1500 g **or** major cardiac anomaly

**Group III **Birth weight less than 1500 g **PLUS **major cardiac anomaly.

Major cardiac anomaly was defined as either cyanotic congenital heart disease that required palliative or corrective surgery or non-cyanotic heart anomaly that required medical or surgical treatment for cardiac failure.

Using the new risk classification scheme survival was 97% Group I, 59% for Group II and 22% for Group III in the 1980's but has improved to 98%, 82%, and 50% respectively in our recent experience [45]. Others have confirmed the value of this classification [46]. A study from Montreal identified only preoperative ventilator dependence and severe associated anomalies as having prognostic significance [47].

### C. Selection for non-treatment

Infants with Potter's syndrome (bilateral renal agenesis) and trisomy 18 which is fatal in the first year of life in over 90% of affected infants should be offered the option of no active treatment. Similarly, infants with totally uncorrectable major cardiac defects or with Grade IV intraventricular haemorrhage should be considered for non-operative management.

### D. Emergency ligation of the distal tracheooesophageal fistula

Generally, the operative correction of an oesophageal atresia is **not **regarded as an emergency procedure. The one exception is the preterm infant with severe respiratory distress syndrome requiring ventilatory support. Ventilatory gases escaping down the distal fistula result in gastric distension which further impedes respiratory function. With progressively increasing gastric distension, the stomach may eventually rupture causing a tension pneumoperitoneum which renders ventilatory support even more difficult [48].

The traditional method of dealing with this sequence of events was to perform an emergency gastrostomy. Unfortunately, most of these infants died because of further dramatic deterioration of ventilation due to the sudden reduction in intragastric pressure allowing free flow of respiratory gases through the tracheooesophageal fistula.

Many manoeuvres have been advocated to alleviate the problem including positioning of the endotracheal tube distal to the fistula. However, if the fistula is sited at the level of the carina, this manoeuvre impossible to achieve. Others have advocated blocking of the fistula by a Fogarty catheter passed at bronchoscopy [49]. The affected infants are usually preterm and in critical respiratory status. The smallest calibre bronchoscope will not permit ventilation while manoeuvring a Fogarty catheter into the distal oesophagus and in a cyanotic infant this will aggravate the condition and further exacerbate the hypoxia.

Since 1984 [50,51], we have advocated emergency transpleural ligation of the tracheooesophageal fistula as the procedure of choice in the infants with combination of problems. Occasionally there is such a dramatic improvement in respiratory status that primary repair of the atresia can proceed. In the majority of cases, ligation of the fistula does improves respiratory status, the thoracotomy is closed pending resolution of the respiratory distress over the subsequent few days. The aim should be to re-operate in 8–10 days to divide the fistula and repair the oesophageal atresia. There is a risk of recurrent fistulisation if there is a prolonged delay following ligation in continuity alone.

#### Operative approach (Fig. 3)

**Figure 3:**
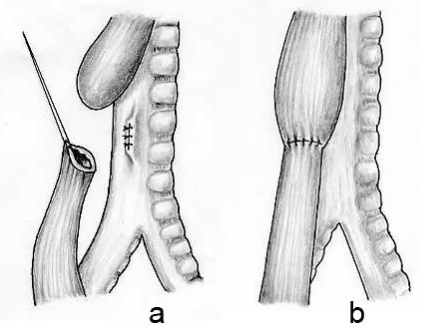
The operative repair of an oesophageal atresia and distal tracheooesophageal fistula.

The operation is performed under general endotracheal anaesthesia with dependable vascular access and employing gentle ventilatory pressure so as not to produce gastric distension.

1. ***Intraoperative endoscopy ***– Preliminary bronchoscopy may be carried out to define the site of entry of the distal tracheooesophageal fistula and to assess the presence of tracheomalacia. An alternative is to carry out an oesophagoscopy to define the length of the upper oesophagus and to exclude an upper pouch fistula which is more common with isolated oesophageal atresia.

2. ***Position ***– The infant is placed on the left side with the right arm across the front of the chest for a right postero-lateral thoracotomy.

3. ***Incision ***– A curved incision centred 1 cm below the inferior angle of the scapula approximately 5–6 cm long is made. The muscle of the chest wall may either be split or divided with electrocautery taking care to preserve the long thoracic nerve supply to serratus anterior. The thorax is opened through the 4^th ^or 5^th ^intercostal space by dividing the intercostal muscles or by entry through the bed of the unresected rib.

4. ***Extrapleural approach ***– has the advantage of conferring protection of the pleural space in the event of an anastomotic leak. Commencing posteriorly, the pleura is gently freed off the chest wall using blunt dissection. The dissection proceeds into the mediastinum to provide good access to the oesophagus. The extrapleural approach is slightly more time-consuming and has theoretically advantages over the transpleural approach still used by many surgeons.

5. ***Exposure of the esophageal segments ***– The azygos vein is the first structure encountered on entering the mediastinum. The azygos vein is gently mobilised and divided between ligatures to expose the esophagus. The distal oesophagus usually lies directly deep to the azygos vein and is identified by the vagus nerve coursing over its anterior aspect. The distal oesophagus can be seen to distend with each inspiration but it is still advisable to gently compress the lumen of the distal oesophagus while the anaesthetist applies increased respiratory pressure. This manoeuvre allows the right lung to expand thus ensuring that the structure compressed is not the right main bronchus. The blind upper oesophageal pouch is identified high up in the mediastinum aided by the anaesthetist applying pressure on the oro- or naso-oesophageal tube.

6. ***Repair of the anomaly ***– The distal oesophagus is mobilised circumferentially just distal to the entry of the tracheooesophageal fistula into the trachea and a soft rubber sling (neoloop) is placed around the oesophagus. A marking seromuscular suture is placed in the lateral wall of the distal oesophagus to assist with orientation. The distal oesophagus is dissected to the level of the fistula and the upper and lower extent of the fistula is marked with fine non-absorbable sutures before dividing the oesophagus just distal to the fistula. The tracheal side of the fistula is closed with interrupted 5.0 sutures to achieve an air-tight closure. The tracheal closure may be tested by instilling warm saline over the suture line while the anaesthesiologist expands the lungs. Having identified the proximal blind oesophageal pouch a figure of eight suture is inserted at its tip to aid in its mobilisation. The proximal oesophagus should be mobilized sufficiently to produce a tension-free anastomosis. Laterally, anteriorly and superiorly the mobilisation is easily achieved, but medially there are fibrous adhesions to the trachea which require sharp dissection. It is important to remain close to the oesophageal wall and avoid entering the trachea during the mobilisation. The end of the upper pouch is now opened to reveal the mucosal surface. An end-to-end anastomosis between the proximal and distal oesophagus is fashioned using interrupted full-thickness fine sutures. The posterior half of the anastomosis is completed first with sutures tied on the mucosal surface. If there is a wide gap, the distal oesophagus can be mobilised safely well down towards the diaphragm. The sutures in wide gap cases should be placed untied in the posterior half of the anastomosis and gently but firmly brought together so as to distribute the tension equally over a larger area. The sutures are then tied sequentially while the tension is maintained. It is almost always possible to achieve an anastomosis when there is a distal fistula present. The anterior half of the anastomosis is completed with interrupted full-thickness sutures. Just prior to the final suture being tied a transanastomotic fine-calibre naso-gastric tube may be passed. This allows gastric decompression in the early postoperative course and provides a route for early enteral feeding.

7. ***Methods to overcome a wide gap ***– Various manoeuvres have been proposed to overcome a wide gap but in our experience a very tense anastomosis can be achieved in most cases and if the infant is subsequently electively paralyzed and mechanically ventilated for approximately 5 days postoperatively, the anastomosis will heal without leakage [52,53]. Others have proposed tubularisation of the upper pouch after creating a flap [54], circular myotomy of the upper pouch [55] or abandoning any attempt at initial primary anastomosis awaiting delayed primary anastomosis 6–12 weeks later [56].

8. ***The thoracotomy incision ***is now closed with or preferably without intercostal drainage especially if the procedure has been totally extrapleural and a technically satisfactory anastomosis has been performed.

*The procedure can be carried out thoracoscopically but this requires advanced skills in minimal invasive surgery. The accumulated results in a multi-institutional review of 104 infants showed comparable outcomes compared with the open thoracotomy procedure *[57].

#### Management of isolated oesophageal atresia

The diagnosis of isolated oesophageal atresia without a fistula should be suspected when on the initial radiograph there is **no **gas in the abdomen ("gasless abdomen"). There is the remote possibility of there being an occluded fistula either by mucus or a complete obstruction [58]. A preoperative bronchoscopy should always be performed in this situation to exclude an upper pouch fistula. The overall incidence of upper pouch fistula in oesophageal atresia is 2.5% but it is 3–4 times more common in isolated atresia.

Once the diagnosis of isolated atresia is made, the next step is to perform a feeding gastrostomy and to estimate the extent of the gap between the proximal and distal oesophagus. The stomach in isolated atresia is very small and it may not be possible to anchor the stomach to the anterior abdominal wall. To avoid tension and necrosis of the stomach it is preferable to leave the stomach at the back of the abdomen and to allow the gastrostomy tube to traverse the peritoneal cavity. The gap between the oesophageal ends is measured by injecting sufficient radioopaque contrast into the stomach to allow it to enter the distal pouch or by passing a bougie (Hegar dilator or urethral sound) through the gastrostomy site into the distal oesophagus. With a radio-opaque catheter in the proximal oesophagus it is now possible to measure, in terms of vertebral body heights, the gap between the two ends. Where the gap is less than two vertebrae, an attempt should be made at immediate primary anastomosis. For a gap of three to six vertebra, delayed primary repair should be planned. Over a period of up to 12 weeks while maintaining suction to the upper pouch and feeding by gastrostomy, the gap gradually narrows.  Regular monitoring of the gap using metal sounds in the distal oesophagus is undertaken. When the two ends can be approximated or overlapped, an attempt at delayed primary anastomosis is undertaken. 

When the gap between the two ends is greater than six vertebrae, the options are to proceed as above and attempt delayed primary repair, to apply graduated tension on the oesophageal ends over a period of 6–10 days and then perform a primary anastomosis [59], or to abandon any attempt to retain the esophagus and to perform a cervical oesophagostomy and replace the oesophagus at a later date.

The incidence of significant gastrooesophageal reflux and the subsequent need for an antireflux procedure is much higher following anastomosis under tension.

#### Management of the H-type fistula

An H-type tracheooesophageal fistula is suspected when the infant experiences coughing with feeds or suffers from recurrent respiratory infections. The diagnosis is established on contrast oesophagogram (ideally a tube oesophagogram) and confirmed at bronchoscopy/oesophagoscopy. It is extremely valuable to have a ureteric catheter passed across the fistula at preliminary bronchoscopy immediately prior to surgery. The operative division of the H fistula is most frequently performed via a low cervical approach.

#### Postoperative management

When the oesophageal anastomosis has been performed under tension, the infant is electively paralysed and mechanically ventilated for five days postoperatively. With this regimen there have been no major disruptions and only a few minor leakages which have healed spontaneously [60]. In all other instances, regular pharyngeal suction is necessary for the first few postoperative days. The suction catheter should be clearly marked to prevent the tube being passed to the site of the anastomosis and causing damage. Transanastomotic nasogastric feeds may be commenced on the second or third postoperative day and when the infant is swallowing saliva, oral feeds may be started. We do not regularly perform a follow-up contrast study but if there is any doubt as to the integrity of the anastomosis a water-soluble contrast study is carried out.

#### Complications

**Early**: anastomotic leakage

anastomotic stricture

recurrent fistula

**Late**: gastrooesophageal reflux

tracheomalacia

dysmotility

**Anastomotic leaks **occur in 15–20% [61] of patients but in only one-third or less is there a major disruption. Major leaks occur in the early postoperative period (<48 hours) and present with life-threatening tension pneumothorax. Emergency treatment consists of tube thoracostomy following which either suction is applied to the intercostal drain while awaiting healing of the anastomosis or an early thoracotomy is carried out with the intention of repairing the anastomosis or, if there has been a complete disruption, abandoning any attempt at re-anastomosis and performing a cervical oesophagostomy and closing the distal oesophagus pending oesophageal replacement.

Minor leaks may be detected on the "routine" contrast study usually performed on the 5–7^th ^day postoperatively. These will all seal spontaneously but there is an increased incidence of later stricture formation.

**Anastomotic strictures **develop in 30–40% [62] of cases most of which will respond to one or two dilatations. Risk factors which have been implicated in stricture formation include anastomotic tension, anastomotic leakage and gastrooesophageal reflux. With meticulous handling of the oesophageal ends, preservation of the blood supply and careful inclusion of mucosa in each and every suture of the anastomosis, strictures can be kept to a minimum.

Endoscopic dilatation of the stricture can be carried out either at rigid oesophagoscopy using semi-rigid bougies (Savary-Gillard) of progressively larger calibre or by balloon dilatation introduced either at fluoroscopy or during flexible endoscopy. In either event, the balloon is carefully positioned under fluoroscopy at the centre of the stricture and contrast is gradually instilled under pressure until the "waisting" at the stricture is abolished. At the end of the procedure, contrast is introduced into the oesophagus to ensure that there has been no perforation and to establish the effectiveness of the dilatation.

Only rarely is it necessary to resort to resection of an intractable anastomotic stricture.

#### Recurrent tracheooesophageal fistula

The incidence of recurrent tracheooesophageal fistula is between 5–14% [63]. A recurrent fistula should be suspected if the infant manifests respiratory symptoms (coughing during feeds, apnoeic or cyanotic episodes) or has recurrent respiratory infections after "successful" repair of the oesophageal atresia. Urgent investigations must be undertaken to positively diagnose or exclude the possibility of a recurrent fistula.

The diagnosis may be suspected on the plain chest radiograph which shows an air oesophagogram. Routine contrast oesophagogram has a low yield and the most useful investigation is a prone tube cine-oesophagogram when water-soluble contrast is slowly instilled into the oesophagus while the nasogastric tube is gradually withdrawn from the stomach to the level of the pharynx.

Bronchoscopic examination will reveal the recurrent fistula at the site of the original tracheooesophageal fistula. It is essential to pass a fine ureteric catheter across the fistula into the oesophagus and to view the catheter in the oesophagus at endoscopy [64]. The passage of a catheter across the fistula is also an essential preliminary step in the operative correction of the recurrent fistula. At surgery it is useful to define the oesophagus above and below the site of the fistula and to insert stay sutures at both the oesophageal and tracheal ends of the fistula before it is divided. The resulting defects in the trachea and oesophagus are closed with firm non-absorbable interrupted full-thickness sutures.

#### Gastrooesophageal reflux (GOR)

GOR is common in all infants following repair of oesophageal atresia – significant reflux occurs in around 40% of cases, about half of whom will require surgical management [65,66]. GOR is more common following anastomosis under tension and after delayed primary repair [67]. It has been implicated in the pathogenesis of anastomotic stricture. However, reflux can still occur following uncomplicated, tension-free anastomosis where the distal oesophagus has remained virtually undisturbed. Incompetence of the lower oesophageal sphincter mechanism may be due to a primary neurgenic disturbance inherent in the development of the oesophageal atresia or to technical factors involved in the repair or it may be due to shortening of the intraabdominal oesophagus and/or abolishment of the gastrooesophageal angle of His.

Symptoms of GOR are similar to those of recurrent fistula with acute or chronic respiratory problems but also include recurrent vomiting and stricture formation. The diagnosis can be established on contrast swallow, pH monitoring and endoscopy and biopsy of the distal oesophagus. Stricture formation at the anastomosis which is resistant to repeated dilatations often resolves spontaneously once the GOR is corrected.

Antireflux medication including gastric acid suppression is only successful in about half the cases. Surgical treatment is problematic given the inherent dysmotility present in the distal oesophagus. A fundoplication may in itself produce a functional obstruction at the gastrooesophageal junction. The failure rate of fundoplication carried out in the first three months of life is excessively high [68]. With regard to the nature of the wrap, there are advocates for partial (Thal) as well as for a short, floppy wrap (Nissen type).

#### Tracheomalacia

Tracheomalacia may be defined as a structural and function weakness of the trachea resulting in partial and occasionally complete respiratory obstruction. The structural abnormality comprises a deficiency in the cartilage in the tracheal rings and an increase in the length of the transverse muscle [40]. The result is that the airway collapses during expiration causing expiratory stridor which varies in severity from a hoarse barking type of cough, to recurrent respiratory infection to acute life-threatening episodes of cyanosis or apnoea. The incidence of tracheomalacia is around 10% and about half will require surgical correction. The area of collapse seen at bronchoscopy is usually restricted to the trachea at the level of the entry of the distal tracheooesophageal fistula, but it can be more extensive. It usually presents within the first few months of life and generally coincides with a period of rapid weight gain. The diagnosis is made at bronchoscopy or cine-bronchography.

If the respiratory obstruction is severe enough to present with acute life-threatening events, treatment needs to be carried out promptly and certainly before the infant leaves hospital [69]. The definitive treatment consists of aortopexy in which the ascending and arch of the aorta are elevated anteriorly towards the sternum. Via a left anterolateral thoracotomy or a median sternotomy, the arch of the aorta is exposed after excising the lateral lobe of the thymus. Three sutures of non-absorbable material are passed several times through the adventitia of the arch and ascending aorta and then through the sternum. The sutures are left untied until all three are in position and then with the assistant depressing the sternum, all the sutures are tied. On releasing the sternum, the aortic arch is pulled forward and in doing so, allows space for the trachea to expand.

The result is usually dramatic with immediate resolution of the obstruction to the air passages [70].

#### Dysmotility

Dysmotility affects the distal oesophagus particularly in relation to abnormal coordination of contractions which in fact can be seen on contrast studies of the oesophagus. The intrinsic innervation of the distal oesophagus has been shown to be abnormal in the fetal rate model and affects both excitatory (SP-labelled) and inhibitory (VIP-labelled) intramural nerves [39]. The dysmotility is a major factor in the long-term swallowing problems encountered in these children [71]. The patients are advised to take fluids liberally with meals and to avoid foodstuffs which exacerbate the problems especially doughy white bread and cakes.

#### Respiratory function

During infancy and for the first three years of life, patients with oesophageal atresia suffer increased frequency of respiratory infections. The respiratory problems tend to resolve with time. The tendency to respiratory infections has variously been attributed to oesophageal dysmotility and/or gastrooesophageal reflux with recurrent aspiration or to a primary respiratory abnormality. Emery and Haddadin [72] suggested that the presence of nonciliated squamous epithelium found in the trachea of 80% of infants with oesophageal atresia could seriously impair the anti-infection mechanism and contribute to repeated attacks of bronchitis. Respiratory function tests carried out in infants soon after repair of the oesophageal atresia have shown abnormal patterns of air flow and also increased airway resistance [73].

**Congenital oesophageal stenosis **due to tracheobronchial remnants in the distal oesophagus in an infant with esophageal atresia is a rare but well documented phenomenon [74,75]. It is thought to arise as a result of defective separation of the trachea from the oesophagus. It may be recognised at the time of the initial repair of the oesophageal atresia when passage of a catheter into the stomach is impeded. Alternatively, symptoms develop quite early with dysphagia and regurgitation of solid food. Contrast radiography shows a short well-defined narrowing in the distal oesophagus which needs to be differentiated from a reflux stricture. Balloon dilatation confirms the short defined narrowing which fails to widen on inflation of the balloon. Endoscopy reveals normal mucosa to the site of the stenosis which, if composed of a complete cartilaginous ring, fails attempts at dilatation. Treatment consists of resection and end-to-end anastomosis. A useful step at the time of surgery is to pass a flexible gastroscope to the level of the stenosis to highlight the precise level of resection as the stenosis is frequently ill-defined from the external surface.

## Oesophageal replacement

The need to replace the oesophagus in oesophageal atresia is extremely rare and should only be considered in very long-gap situations or where repeated attempts at retaining the host oesophagus have failed and the infant's survival is at risk. A full discussion of oesophageal replacement is beyond the scope of this review. There are basically three methods of oesophageal replacement currently being practiced in children – gastric transposition [76], colonic interposition [77] and jejunal interposition [78]. Each have merits and carry complications and it is incumbent on the individual surgeon to be proficient in one technique to achieve the optimal results.
